# Investigation of Delamination and Cutting Force in the Drilling of AA7050-CFRP Hybrid Composite Stacks Used in Real Aerospace Applications

**DOI:** 10.3390/polym18080938

**Published:** 2026-04-11

**Authors:** Furkan Taşcı, Abdülcelil Bayar, Ali Yüksel, Çağlar Yavaş, Çağın Bolat, Ali Ercetin, Fatih Akkoyun, Bekir Yalçın

**Affiliations:** 1 Department of Mechanical Engineering, Afyon Kocatepe University, 03200 Afyonkarahisar, Türkiye; furkantc6@gmail.com (F.T.); aliiyuksel@yandex.com (A.Y.); 2 Turkish Aerospace (TUSAŞ), 06980 Ankara, Türkiye; celilbayar@gmail.com; 3 Karcan Cutting Tools, 26110 Eskişehir, Türkiye; c.yavas@karcan.com; 4 Department of Mechanical Engineering, Engineering and Natural Sciences Faculty, Samsun University, 55400 Samsun, Türkiye; cagin.bolat@samsun.edu.tr; 5 Department of Naval Architecture and Marine Engineering, Maritime Faculty, Bandırma Onyedi Eylul University, 10200 Bandırma, Türkiye; 6 University College, Korea University, 145 Anam-ro, Seoul 02481, Republic of Korea; 7 Department of Mechanical Engineering, University of Izmir Demokrasi, 35140 Izmir, Türkiye; fatih.akkoyun@idu.edu.tr

**Keywords:** laminated structures, dry drilling, delamination, thrust force, fiber-reinforced polymers

## Abstract

This study investigates the combined effects of drilling parameters and tool features on thrust force during drilling of hybrid AA7050/CFRP stacks intended for aerospace applications. Experiments were designed using a Taguchi L16 (4^2^2^2^) orthogonal array by considering feed rate, cutting speed, drill diameter, and coating condition. Unlike most previous studies on Al/CFRP stack drilling, this work provides a layer-specific evaluation by separately analyzing the thrust-force responses of the AA7050 and CFRP layers, while also correlating force behavior with aluminum-side damage and CFRP-side delamination within a unified framework. For the aluminum layer, feed rate was the dominant factor affecting thrust force, with a contribution of 71.22%, followed by drill diameter (26.22%). For the CFRP layer, drill diameter was the most influential parameter (52.58%), followed by feed rate (43.77%). Thrust force ranged from 155.24 to 414.45 N for AA7050 and from 77.73 to 175.39 N for CFRP. Uncoated tools and higher feed rates increased thrust force, leading to poorer machining performance. Delamination in the CFRP layer was primarily governed by feed rate (46.71%), followed by drill diameter (35%). Overall, increasing feed rate and drill diameter significantly deteriorated the machinability of the hybrid structure.

## 1. Introduction

Sustainability-driven industrial transformation necessitates the integration of next-generation material solutions with mechanical engineering design principles. Currently, objectives such as reducing carbon footprints, optimizing life cycle performance, and minimizing resource utilization prioritize environmentally friendly approaches in both material selection and manufacturing methodologies. In this context, advanced composites with high strength-to-weight ratios, nanostructured hybrid materials, biodegradable polymers, and recyclable metallic alloys offer the potential to synergize lightweight design and energy efficiency. Lightening yields significant advantages, such as lower energy consumption, reduced actuation power requirements, and improved dynamic response, by decreasing the average mass in mechanical systems. Consequently, strategies involving geometric/topology optimization, composite configurations, and functionally graded materials for structural components are rapidly proliferating across various sectors, ranging from automotive and aerospace to robotics and renewable energy systems. Energy efficiency is directly correlated not only with the material itself but also with the digitalization of manufacturing processes, the adoption of low-waste technologies such as additive manufacturing, and the planning of post-use recycling pathways. The adaptability to diversified conditions, sensor integration capability, and multifunctionality provided by next-generation materials facilitate smart system design while enabling a more systematic approach to sustainability objectives. In particular, materials that align with green manufacturing principles and reduce operational energy consumption contribute to the development of environmentally and economically competitive solutions in mechanical engineering. In conclusion, when evaluated from a sustainability perspective, lightening, energy efficiency, and innovative material selection constitute the fundamental components not only of meeting environmental obligations but also of developing high-performance and durable engineering systems.

The concept of composites has evolved over time to include diverse material combinations, such as metals and polymers, in order to strike a balance between high strength requirements and ecological concerns aimed at reducing fuel consumption [1,2]. The primary reason for the widespread utilization of Carbon Fiber-Reinforced Polymers (CFRP) is their high strength-to-weight ratio; owing to these properties, CFRP is frequently preferred in the aerospace and automotive industries [3,4,5]. Beyond the machinery and manufacturing sectors, these materials are also employed in the construction and architectural fields for structural reinforcement and lightweight restoration applications [6]. Furthermore, CFRP materials are applied in large-scale wind turbine blades, ensuring that interest in low-mass renewable energy systems is maintained [7,8]. Nevertheless, CFRP structures exhibit certain drawbacks, such as low bearing strength, which compromises impact resistance. To overcome these limitations, metal/CFRP hybrid composites, which combine the formability of metals with the high specific strength of CFRP, have been developed as an alternative solution [9,10]. Specifically, the integration of aluminum alloys with CFRP enables the production of Al/CFRP hybrid composites with superior mechanical properties by synergizing the advantages of both materials [11].

Bolting and riveting are widely utilized joining methods, particularly for aerospace materials. However, the reliability and quality of these joints depend heavily on the quality of the holes produced prior to the assembly process. The quality of holes drilled in composites is characterized by parameters such as roundness, cylindricity, waviness, and hole surface roughness. Herein, poor hole quality can lead to failure by inducing high stress concentrations in riveted joints. Simultaneously, machining-induced defects such as delamination and micro-cracks significantly reduce the performance of composites [12]. In addition to these issues, the process becomes susceptible to hybrid effects, particularly in laminated structures consisting of composite/metal or metal/polymer stack combinations [13]. For instance, for the AA7075 and CFRP combination, various challenges arise during the simultaneous drilling of two materials with distinct chemical and physical properties. Aside from the possible handicaps such as fiber breakage, fiber pull-out, delamination, and matrix thermal degradation in the CFRP material, chip evacuation and built-up edge (BUE) formation on the main cutting edge are among the most critical problems encountered in AA7075 [14]. In the context of metal/composite hybrid systems, chip evacuation and chip morphology represent other significant factors influencing cutting forces and delamination. While metallic layers tend to produce continuous or serrated chips, powdery and discontinuous chips are generated from CFRP layers [15,16]. Furthermore, depending on the stacking sequence of the metal and CFRP layers, the efficient evacuation of chips originating from the previously drilled layer can mitigate the risk of chip clogging in the overall system.

Drilling is one of the critical operations in machining and is influenced by a multi-layered set of inputs, including process, material, tool, and lubricant. High cutting forces generated during drilling lead to energy loss and tool wear [17,18]. In contemporary aerospace applications, diverse material combinations comprising composites and aluminum and/or titanium are utilized across a wide range of structures, such as wings or tail planes. Such structures contain holes for various purposes, including bolt holes [19]. In aerospace manufacturing, the drilling of multi-material stacks is one of the most commonly employed machining processes during aircraft assembly [20]. Even though abrasive water jet machining can be an effective solution for trimming the edges of composites and other materials, hard cutting tools and multi-step drilling methods are generally required when hole making is involved in such material stacks [21]. Drilling operations performed on computer-controlled and multi-axis machine tools still remain the most frequently used methods in many sectoral implementations. Both R&D engineers and scientific researchers endeavor to achieve maximum cutting efficiency through the optimization of process parameters. At this juncture, input variables such as fiber/matrix pairing of the composite design, fiber ratio, fiber type (e.g., glass, carbon, and basalt), number of layers, interlaminar adhesive condition, lubricant usage, and metal/composite pairing constitute the key factors specific to the problem.

Regarding tool-oriented solutions, standard drill bits, which are easily commercially available from cutting tool suppliers, are generally preferred by primary manufacturers. However, conventional twist drills are subjected to rapid wear during cutting due to the drastically different phase properties of the composite-metal laminate, leading to a deterioration in hole surface quality. When options with peripheral cutting edges, such as brad drill tools, are employed, average cutting forces tend to exhibit an increasing trend [22]. In addition, the stringent quality requirements demanded in aircraft component manufacturing obligate the frequent replacement of drill bits, resulting in additional costs for manufacturers [23]. In recent years, particularly with advancements in tool technologies, cutting forces have been reduced without compromising delamination by using specially designed drilling tools with varying point angles, web thicknesses, and modified body geometries [24]. On the other hand, upon examining certain studies, significant reductions have been achieved in total consumed cutting energy, tool wear, and measured average cutting torque values, attributable to design- and coating-oriented trends (such as TiAlN or diamond) observed in tool technologies [25,26].

In this study, utilizing a hybrid-designed workpiece composed of aerospace-grade aged AA7050 and CFRP layers, the optimization of cutting forces and delamination for contemporary aerospace products has been carried out. This alloy was specifically selected because AA7050-T7451 is widely associated with highly loaded aerospace structural components where a combination of high specific strength, fracture toughness, and improved stress corrosion resistance is required. Therefore, compared with alternative aerospace alloys such as AA2024 and AA7075, AA7050-T7451 was considered to provide a more suitable metallic counterpart for representing demanding service-oriented hybrid stack configurations. To this end, to draw a comprehensive perspective, the combined effects of main process parameters and tool design features were investigated experimentally and analyzed quantitatively using statistical Design of Experiments (DoE) techniques. The primary novelty of this study lies in the integrated and layer-specific evaluation of drilling performance in an aerospace-grade AA7050-T7451/CFRP hybrid stack. Unlike many earlier studies that focused mainly on overall stack behavior or a limited set of output responses, the present work separately quantifies thrust-force behavior in the Al and CFRP layers under the same drilling conditions. In addition, the study jointly evaluates the effects of feed rate, cutting speed, drill diameter, and coating condition not only on thrust force but also on two distinct damage metrics, namely the damage factor for the Al layer and the delamination factor for the CFRP layer. This combined approach offers a more application-oriented understanding of hybrid-stack machinability for real aerospace assemblies. The results obtained will shed light not only on the assembly of civil- and military-oriented aircraft components but also on ensuring the expected joint strength, delamination resistance, and dimensional precision for future assembly applications in sectors such as renewable energy, automotive, and construction involving metal/CFRP/metal sandwich structures.

## 2. Materials and Methods

In this study, the aim is to characterize the drilling process and optimize process parameters in hybrid laminates composed of aluminum (AA7050) and carbon fiber-reinforced polymer (CFRP) layers. Accordingly, the experimental parameters were determined as feed rate at four levels (0.05, 0.075, 0.1, 0.15 mm/rev) and cutting speed at four levels (15, 30, 45, 60 m/min). For the tool variables, the drill diameter was set at two levels (4 and 6 mm), and the coating condition was established at two levels (coated and uncoated drill) (Table 1). Drilling force and delamination analysis were conducted as output variables, and the Taguchi L16 (4^2^2^2^) orthogonal design method was applied to the relevant results (Table 2). The selected factor levels were determined based on a combination of practical aerospace drilling relevance, tool manufacturer specifications, and the need to cover a sufficiently wide but stable operating window for the Taguchi design. The feed-rate levels were chosen to represent low-to-moderately high chip-load conditions commonly encountered in the drilling of hybrid composite stacks, while allowing the sensitivity of thrust force and delamination to be tracked progressively. The cutting-speed levels were selected to span a practical dry-drilling range without causing excessively unstable thermal conditions. The drill diameters of 4 and 6 mm were chosen as two representative hole sizes relevant to aerospace fastening/joining applications, while the coated/uncoated comparison was included to evaluate the effect of a commonly used industrial coating condition under otherwise identical tool geometry. Thus, the selected levels were intended to provide both practical relevance and statistical comparability within the experimental framework.

In this study, an aluminum–carbon fiber-reinforced polymer (Al–CFRP) hybrid laminate structure was employed to represent structural configurations frequently encountered in the aerospace sector. The test specimen, which was subjected to grinding with 400, 800, and 1000 grit sandpapers, was created with AA7050-T7451 aluminum alloy for the upper layer owing to its low density, heat treatability, and high specific strength. The AA7050 aluminum alloy was selected to provide a balanced combination of mechanical strength and fracture toughness [27]. Furthermore, this Al–Zn alloy is frequently preferred for internal aircraft structures, such as wing and fuselage attachments. However, the AA7050 alloy is susceptible to intergranular stress corrosion cracking in certain tempers, such as the T6 temper, when in contact with chlorides [27]. The T7 temper enhances stress corrosion cracking (SCC) resistance; therefore, the 7050 series offers significant advantages in this condition.

Carbon fiber-reinforced epoxy composites with 2 × 2 twill weave architecture were utilized in this study. The laminate was fabricated by stacking 33 plies in the specified lay-up sequence and subsequently cured according to the manufacturer’s recommended curing conditions. The nominal cured ply thickness was 0.31 mm, resulting in a total laminate thickness of 10.23 mm. All drilling experiments were performed on fully cured laminates, and specimens were prepared to ensure consistent thickness and material integrity prior to machining. The total thickness of the CFRP layer was 10.2 mm, while the aluminum layer was 6 mm (±0.15 mm), resulting in a total stack thickness of 16.2 mm. According to the supplier information, some critical properties of the matrix resin and carbon fibers can be found in Table 3 and Table 4, respectively.

**Figure 1:**
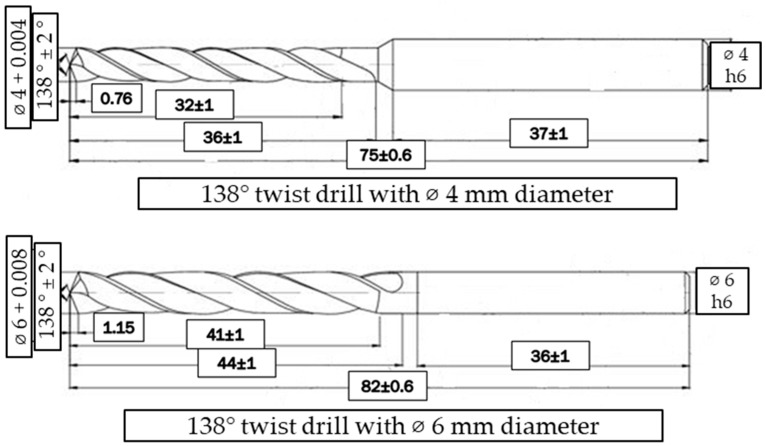
Technical dimensions of the utilized drill bits.

Repeated drilling operations were carried out for each experimental condition using a new tool for that specific condition. The mean values of all results were calculated for both cutting force and delamination and were subsequently utilized for the Analysis of Variance (ANOVA). Drilling operations were conducted on a Hartford VMC-1020 CNC, (Hartford manufacturer from Taichung, Taiwan) vertical machining center (*X*-axis motion: 1020 mm, *Y*-axis motion: 510 mm, *Z*-axis motion: 400 mm, maximum spindle speed: 6000 rev/min) located at the High Technology Application and Research Center of Isparta University of Applied Sciences. WC-Co tungsten carbide base drills with a 138–point angle (supplied by Karcan Cutting Tools, Eskişehir, Türkiye) were selected as the cutting tools, and all operations were executed under dry cutting conditions (without the use of coolant). The technical drawing details and certain critical mechanical/physical properties of the cutting tools with varying diameters are presented in Figure 1 and Table 5.

**Figure 2:**
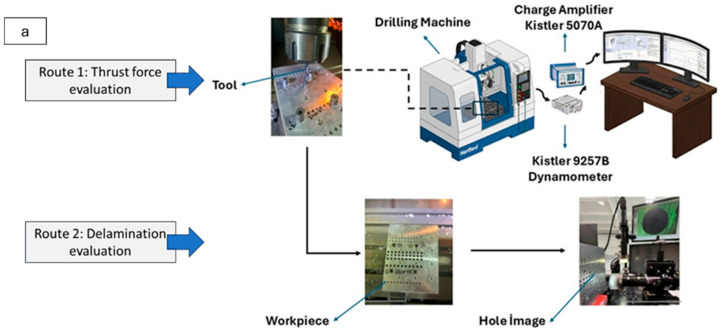

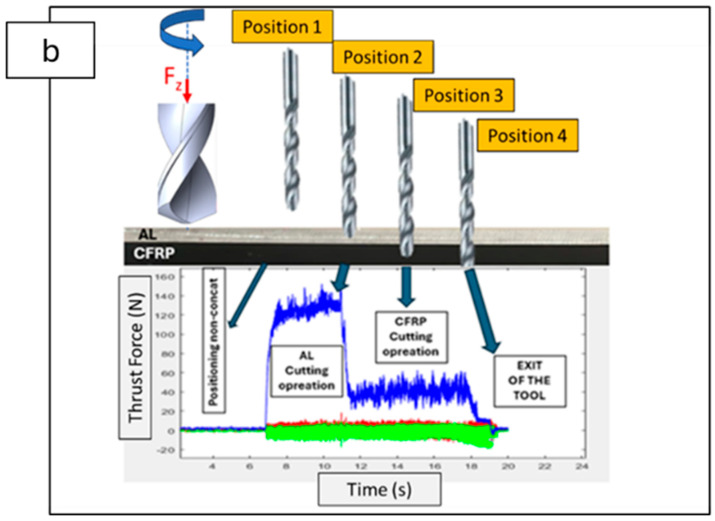
Real views of the utilized CNC equipment and hole image analyzer (**a**) and force measurement strategy for hybrid stacked workpieces (**b**).

The axial force (F_z_) generated during drilling was measured using a Kistler 9257B piezoelectric three-component dynamometer (Winterthur, Switzerland) connected to a Kistler 5070A charge amplifier. The dynamometer recorded the forces generated during the cutting process between the drill bit and the workpiece with high temporal resolution (at the millisecond level) and transmitted the data to a computer via the signal amplifier. Approximately 24,000 samples were recorded for each drilling operation, and these data were processed using MATLAB software (version R2018a). The raw force data were converted into force-time graphs, and the maximum axial force (F_max_) values were identified for each hole. Due to the Al–CFRP hybrid plate structure, drilling forces were evaluated separately for the AA7050 and fiber-reinforced composite regions. Statistical analyses were performed on the obtained mean force data. The effects of different drilling parameters on thrust force were determined through Analysis of Variance (ANOVA), percent contribution ratios, and graphical evaluations. This ensured a more accurate and detailed characterization of the forces occurring within the hybrid material structure. The detailed flowchart of the machining process and the methodology for cutting force determination are presented in Figure 2a,b.

## 3. Results and Discussion

### 3.1. Thrust Force Analysis

**Figure 3:**
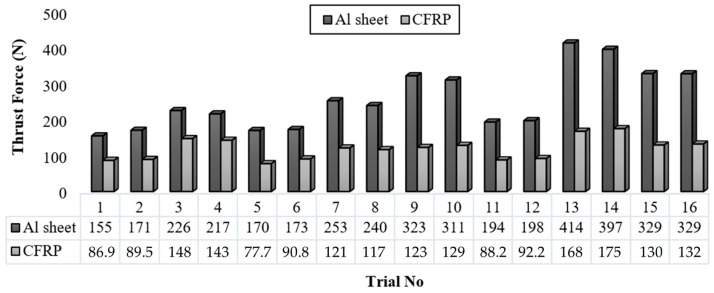
Thrust force levels of the tested samples for each trial.

In the drilling operation, the cutting tool initiates deformation by first machining the metallic layer, with the initial contact surfaces concentrated on the chisel edge and the rake face. Subsequently, the tool penetrates the CFRP layer, completing the drilling process. In this experiment, distinct variations in drilling force emerged, differing from phenomena observed in conventional homogeneous materials, as the metal and composite structures were machined sequentially within a single process cycle. Figure 3 illustrates the distribution of drilling force values obtained for the AA7050-T7 and CFRP layers resulting from drilling operations performed on the Al–CFRP hybrid structure. Upon examination of the graph, it is observed that different combinations of drilling parameters induce significant variability in the axial forces exerted on both material layers. In a similar vein, a study conducted on Al/CFRP stacks reported that thrust force values during the drilling of the Al plate were approximately twice as high as those for CFRP and exhibited a sudden increase as the drill entered the Al plate; this was attributed to the abrupt change in hardness of the Al plate relative to the CFRP [28].

While the maximum force values measured in the aluminum layer ranged between approximately 150 N and 400 N, these values occurred within the 80–190 N range for the CFRP layer. Particularly under certain experimental conditions (e.g., experiments 13 and 14), high thrust force values were recorded for both layers. Factors such as high feed rate and increased tool diameter influenced this result. While the phenomenon of simultaneous contact surface area expansion due to increased tool diameter constitutes one of the causes of this situation, the initial wear deformation of the tool, heated by friction following hard metal cutting, may also have contributed to these results by showing an increasing trend combined with high feed levels. Furthermore, the fact that the tools used in these conditions were uncoated increased the wear resulting from cutting in the metallic region. Conversely, the low force values observed in trials 1 and 5 indicate that the cutting conditions applied in these experiments were selected within a more controlled and appropriate range. When compared with parallel literature studies, average values of 155 N for the Al section and 77 N for the CFRP section were achieved in this study, and the results can be defined as highly competitive. In a study conducted on an Al/Ti/CFRP stack, it was reported that average forces were 135 N for CFRP, 400 N for titanium, and 250 N for aluminum, and that drilling force increased with increasing feed and cutting speed [1]. In another study, the drilling force recorded during Al drilling was approximately twice as high as that for CFRP drilling, measured as 286 N in Al and 143 N in CFRP [29].

### 3.2. Thrust Force Model for Metallic Layers

In Table 6, the effects of four fundamental process parameters (feed rate, cutting speed, drill diameter, and coating condition) on the drilling force were investigated using Analysis of Variance (ANOVA). This analysis evaluated the contribution of each variable to the total variance and its statistical significance. In this study, the R-sq value was determined to be 99.40%. This value demonstrates that the level selection ranges in the study and the obtained parameter effect analyses are significant. The contribution ratio table for all parameters and the corresponding *p*-value results are presented in Table 7.

According to the Analysis of Variance (ANOVA) results presented in Table 5, it was determined that the feed rate was the most influential parameter on the maximum drilling force measured in the Al layer (Delta ranking: Feed Rate > Drill Diameter > Coating > Cutting Speed). The contribution ratio of the feed rate was calculated as 71.22%, with a *p*-value of 0.0000001224. This value is well below the 5% significance level, indicating that the feed rate has a highly significant and determinant impact on the forces generated during drilling. Similar findings have been reported in the literature. Studies have stated that high feed rates are not suitable for drilling Al–CFRP stacks [30]. Bonhin et al. reported that drilling force increased with increasing feed rate in experiments conducted on fiber–metal laminates using Ø4.8 mm coated carbide drills [31]. Zhao et al. supported the same result in their study conducted on Al–CFRP hybrid structures [32]. In experiments conducted on 2024-T351 alloy with different drill diameters and feed rates, Craciun et al. demonstrated that an increase in feed rate significantly raised the drilling force [33]. Similarly, Uddin et al. reported that increasing the feed rate significantly increased the drilling force in their study on Al 6061 alloy [34]. The increase in feed rate is also correlated with tool design, which may reduce indentation effects in twist drills with large point angles. Consequently, the compromised penetration capability may have been expended as cutting energy, potentially triggering an increase in average cutting forces.

The second most influential parameter was the drill diameter, with a contribution ratio of 26.22%. The *p*-value associated with the drill diameter is 0.0000004868, indicating that this parameter was also statistically highly significant. As the diameter increases, the contact area between the tool and the workpiece expands, leading to an increase in the cutting load and, consequently, the generated drilling force. Furthermore, with smaller tool diameters, indentation effects act alongside cutting deformation, but this double impact decreases with increasing tool diameter, and cutting forces increase due to the expanding plastic deformation area. This phenomenon is consistent with the results observed in studies conducted by Kyratsis et al. [35]. The contribution ratio of the cutting speed remained limited at 1.07%, and the *p*-value was calculated as 0.0544. This value lies just above the 5% significance level, suggesting a marginally significant effect from a statistical perspective. Therefore, the effect of cutting speed should not be disregarded; however, it should be noted that it holds lower importance compared to other parameters. The lowest contribution ratio belongs to the coating condition at 0.89%. Although its effect appears limited, the *p*-value of 0.0145 demonstrates that this variable is statistically significant. Overall, while geometric and mechanical parameters such as feed rate and drill diameter exhibit a dominant effect on thrust force, secondary parameters such as cutting speed and coating condition provide more limited yet statistically significant contributions.

**Figure 4:**
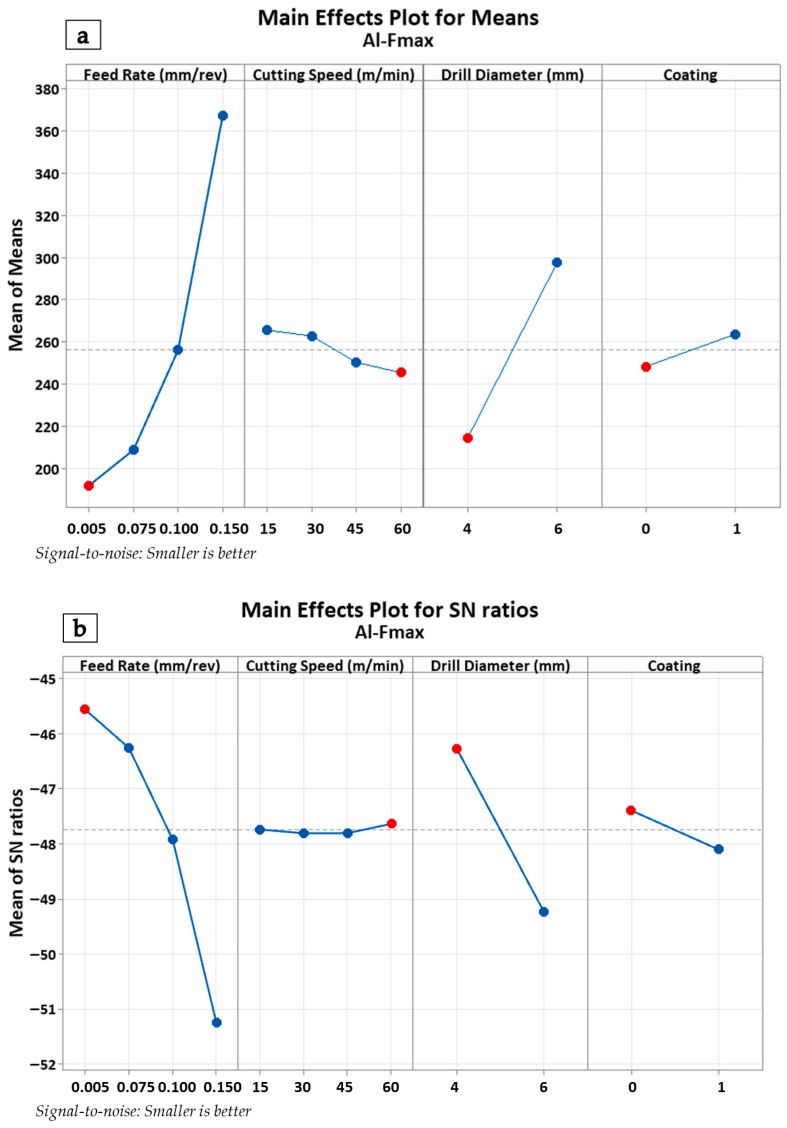
Main effects plots of metallic layers for thrust force values (**a**) and SN plots of metallic layers for thrust force values (**b**).

The main effects plot in Figure 4a indicates that feed rate and drill diameter have a pronounced effect on thrust force. A substantial increase in drilling force was observed as the feed rate increased from 0.050 mm/rev to 0.150 mm/rev. In particular, a sharp surge occurred within the 0.1 mm/rev to 0.15 mm/rev range. Similarly, increasing the drill diameter from 4 mm to 6 mm was identified as another factor contributing to the rise in drilling force. Conversely, it was observed that cutting speed exerted a less significant influence on thrust force within the 15–60 m/min range. Regarding the coating condition, it was concluded that coated drills (1) were relatively less effective in reducing drilling force compared to uncoated ones (0). In the Signal-to-Noise (S/N) ratio analysis shown in Figure 4b, the ‘smaller-is-better’ principle was adopted. This analysis revealed that feed rate and drill diameter not only increased the drilling force but also significantly influenced process variation. On the other hand, the coating condition enhanced process stability by improving the S/N ratios. These findings indicate that the use of coated drills can enhance the consistency of the drilling operation and constitute a critical parameter for process optimization. The Anderson–Darling normality test was applied to determine whether the drilling force data followed a normal distribution. The obtained dataset (*N* = 16) exhibited conformity to a normal distribution with a mean of 256.2 N and a standard deviation of 83.51 N (*p*-value: 0.120). The validation of the normality assumption enabled the reliable application of parametric tests and Analysis of Variance (ANOVA).

### 3.3. Thrust Force Model for CFRP Layers

In Table 8, the effects of four fundamental process parameters (feed rate, cutting speed, drill diameter, and coating condition) on the drilling force specific to the CFRP layer were investigated using ANOVA. This analysis evaluated the contribution of each variable to the total variance and its statistical significance. In this study, the R-sq value was found to be 98.99%. Additionally, according to the data obtained in Table 9, the drill diameter exhibited the highest effect, with a contribution ratio of 52.58%. The second most influential parameter was the feed rate with a contribution ratio of 43.77%, which is statistically highly significant (*p* < 0.001). The coating condition ranks as the third most influential variable, with a contribution ratio of 1.38% (*p* = 0.017). In contrast, cutting speed did not show a significant effect, with a contribution ratio of 1.24%. Overall, it was concluded that the most effective parameters on the thrust force were drill diameter and feed rate, respectively, while the coating condition contributed to a certain extent and the cutting speed had a limited effect.

**Figure 5:**
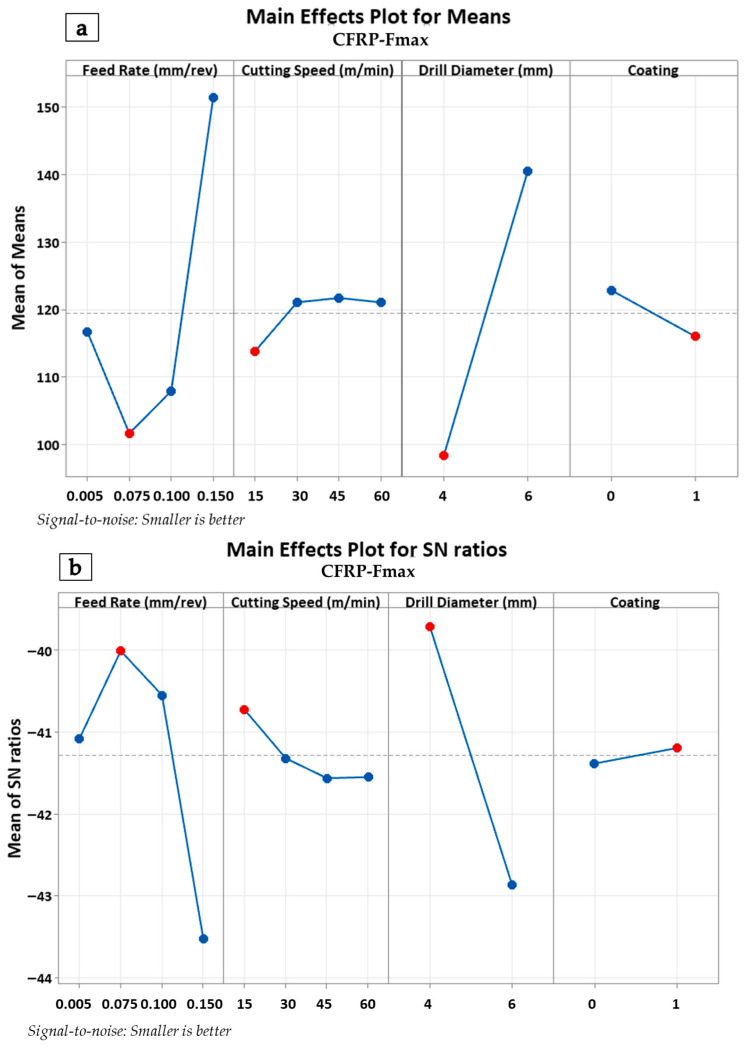
Main effects plots of CFRP layers for thrust force values (**a**) and SN plots of CFRP layers for thrust force values (**b**).

In Figure 5a, the feed rate stands out as one of the parameters with the most pronounced effect on the drilling force. A sharp increase in drilling force was observed as the feed rate increased from 0.050 mm/rev to 0.150 mm/rev. This indicates that higher feed rates lead to greater cutting resistance and, consequently, higher drilling forces during the drilling of the CFRP material. Being in contact with a larger volume of material along the cutting path per revolution is one of the reasons for this phenomenon. Furthermore, it should not be overlooked that fiber reinforcements exhibit a greater bending response following high feed rates. The drill diameter had the most significant impact on the drilling force, and a significant rise in thrust force was observed as the diameter increased from 4 mm to 6 mm. This revealed that larger diameter drills require higher drilling forces due to increased material removal and the adhesion of the epoxy matrix, heated in the tool/workpiece and tool/chip contact zones, onto the tool. Regarding cutting speed, it was determined that cutting speeds ranging from 15 to 60 m/min had a limited effect on the drilling force. This result suggests that cutting speed plays a less critical role compared to other parameters during the CFRP drilling process. Generally, for monolithic CFRP components, there is a tendency for force reduction due to friction-induced thermal softening of the workpiece associated with increasing cutting speeds. This phenomenon was observed to some extent, particularly at low feed rates; however, dynamic process variables such as the evacuation of additional chips resulting from the prior drilling of the metal layer and the initial wear of the cutting tool constrained the influence of this variable. Regarding the coating condition, it was revealed that coated drills (status = 1) were more effective in reducing drilling force compared to uncoated ones (status = 0). This situation can be attributed to the relatively higher friction-induced local softening effects in uncoated tools than in coated ones. Hardness in the hot environment is a critical indicator for these results. The analysis conducted with the ‘smaller-is-better’ principle in Figure 5b indicates that feed rate and drill diameter not only increase the thrust force but also significantly affect process variation. The coating condition enhanced process stability by improving the S/N ratios. This demonstrates that coated drills play a significant role in improving the consistency of the drilling operation.

**Figure 6:**
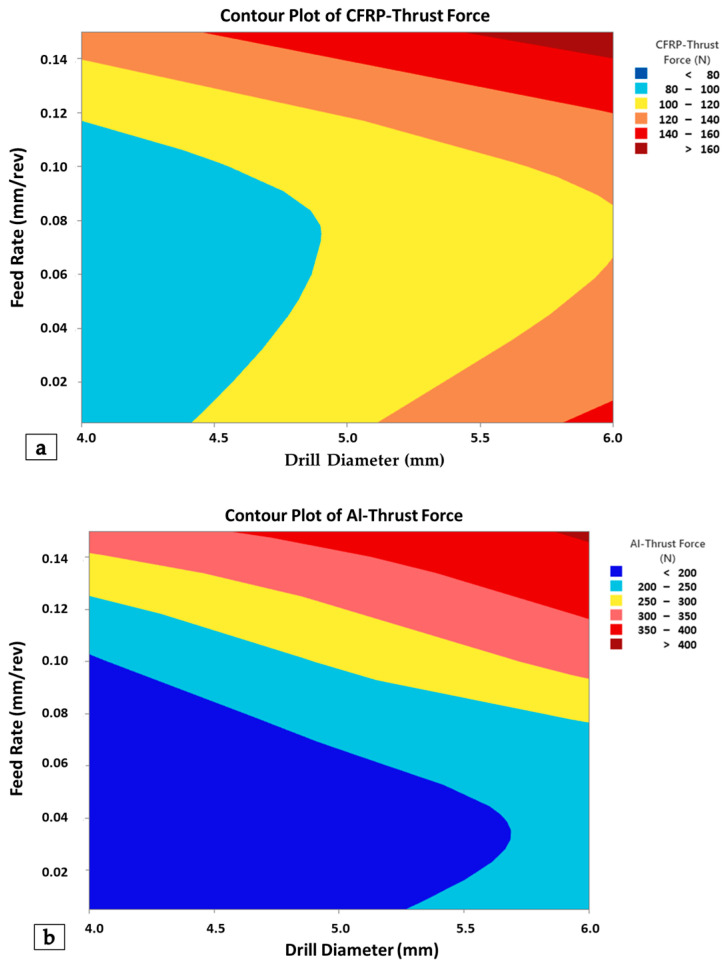
Contour graphs for CFRP (**a**) and Al (**b**) layers depending on feed rate and drill diameter.

In Figure 6, contour plots illustrating the two-way interaction relationships are presented for both the aluminum and CFRP layers. Feed rate and tool diameter, identified as the most influential parameters for both layer components, were designated as the main axis indicators. Here, while low feed rates and small tool diameters stood out as indicators minimizing cutting-induced forces for both CFRP and Al layers, it was observed that the regional transition zones associated with force increase were broader in the CFRP material; in other words, the cutting force increased at a lower rate compared to the Al plate.

### 3.4. Damage Factor for the Al Plate

**Figure 7:**
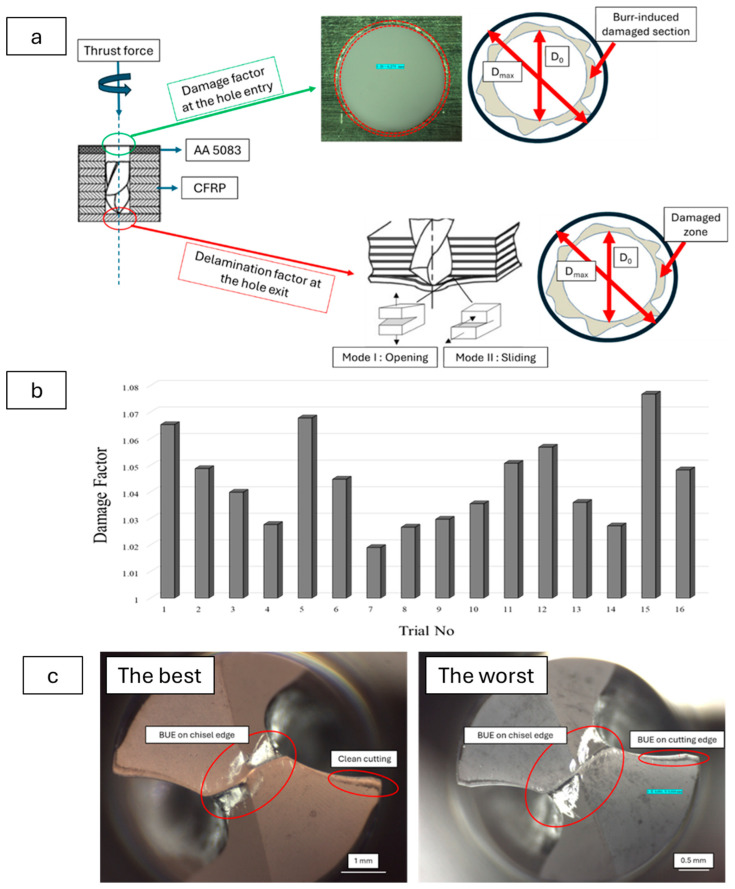
Drilling-induced delamination mechanism and calculation variables (**a**), damage factor results for each Taguchi trial (**b**), and OM views of the best and worst damage factor results according to drill bit condition (**c**).

Drilling-induced damage mechanisms associated with the composite system were evaluated separately for both the Al top plate and the CFRP bottom plate. For the metallic surfaces, particularly in the zone adjacent to plastic deformation at the hole entry, peripheral burr adhesion and local peeling effects were analyzed using the damage factor approach. As the CFRP plates were located at the hole exit, delamination factor analyses were conducted on the exit side. For these analyses, the damage factor was calculated for both layers as the ratio of the maximum damage diameter to the nominal hole diameter. A schematic illustration of the relevant method is presented in Figure 7a.

According to Taguchi trials (Figure 7b) and ANOVA results (Table 10), the most decisive parameter for the damage factor is the drill diameter, explaining 70.88% of the total variance (*p* = 0.001). This indicates that the tendency for damage around the hole significantly increases with the increase in drill diameter. As the diameter increases, the contact area between the tool and the material expands and average cutting forces rise, leading to plastic deformation and micro-crack formation on the bottom surface of the Al layer. The contribution ratio of the cutting speed is 7.72%, which was not found to be statistically significant (*p* = 0.455). However, the rise in cutting temperature associated with increased speed may cause hole softening and local plasticization in the Al layer; this can be considered a mechanism that indirectly affects the damage factor. The contribution of the feed rate was 3.02% (*p* = 0.768), and this parameter was also not statistically significant. The reason for this is that due to its ductile structure, the Al layer does not exhibit delamination behavior as sensitive as that found in fiber-reinforced composites. The contribution ratio of the coating condition is very low (*p* = 0.985), with no significant effect on the damage factor. This result demonstrates that damage formation around the hole on the Al layer is primarily associated with tool geometry and the diameter parameter and that surface coating does not play a determinant role. Additionally, the relatively high damage observed at smaller diameters (4–4.5 mm) may be due to BUE or local singularities created by the tip geometry on the bottom plate. At small diameters, the cutting force density may be higher, and, since the chip flute is narrower, the BUE effect may be reflected onto the Al surface/chip-metal interface. The lower hole-periphery deformation at medium diameters (around 5 mm) can be explained by more favorable chip evacuation and a more homogeneous distribution of cutting forces. In the optical microscope (OM) analyses provided below, the distinct interaction between the BUE effect and drill diameter is also demonstrated specifically for the lowest and highest damage factors (Figure 7c).

**Figure 8:**
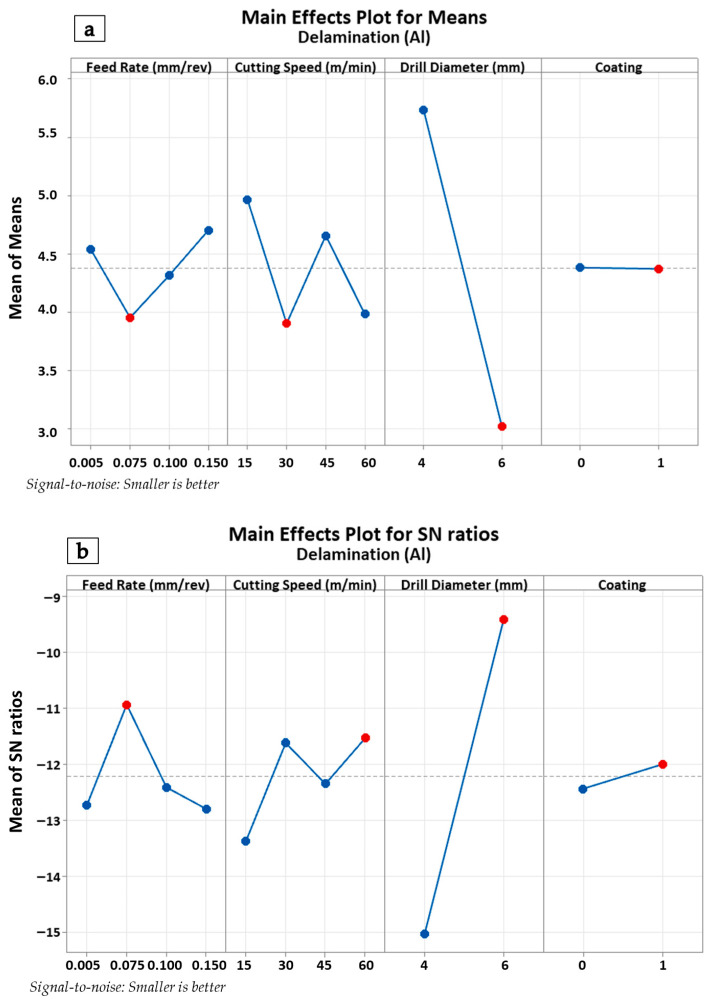
Main effects plots of damage factor levels for metallic layers (**a**) and SN plots of metallic layers for damage factor (**b**).

The main effects and S/N plots presented in Figure 8a,b indicate that the most determinant effects on the damage factor are obtained with feed rate and drill diameter. A remarkable increase in the damage factor level within the Al layer was observed as the feed rate was increased up to 0.150 mm/rev. Similarly, decreasing the drill diameter from 6 mm to 4 mm resulted in a significant rise in the extent of the damage factor. It was observed that the cutting speed parameter had a limited effect on delamination within the 15–60 m/min range. Upon examining the coating factor, it was determined that there was no distinct difference between coated and uncoated tools in terms of delamination levels, and that the coating did not provide any significant improvement in delamination in the Al layer.

It is observed that the vast majority of data points lie around the reference line and remain within the confidence interval limits. The Anderson–Darling (AD) statistic of 0.282 and a *p*-value of 0.590 indicate that the delamination data satisfy the normal distribution assumption (*p* > 0.05). This result confirms that there is no significant deviation in the experimental data and validates that the Analysis of Variance (ANOVA) can be applied reliably. With a mean delamination value of 4.378 and a standard deviation of 1.668, the data exhibit a reasonable dispersion. Overall, the plot reveals that the delamination values in the Al layer exhibit a statistically normal distribution and that the obtained results are consistent with the model.

### 3.5. Delamination Analysis for the CFRP Plate

There are many approaches (diameter-based, area-based, hybrid equations, and image-based) to measure the delamination factor in the technical literature, but the diameter-based approach was preferred due to its ease of application and the ability to make rapid comparisons between samples. Additionally, it does not require any additional digital image processing or secondary operations, thereby providing a quick comparison between the drilled samples under the same cutting conditions. As such, diameter-based damage analyses were preferred, as also adopted by other research teams [36,37,38] focusing on delamination of fiber-reinforced composites.

**Figure 9:**
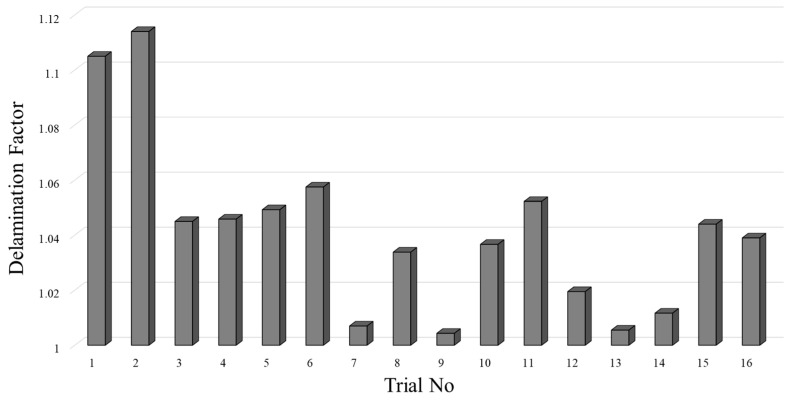
Delamination factor results of each Taguchi trial for CFRP layers.

Push-out delamination emerges around the hole exit periphery of the CFRP plates and induces additional mechanical stress in the bending direction. Particularly, opening stresses in the form of Mode I and the accompanying Mode II shear stresses reflect the primary triggering factors for this type of damage. Since the metallic plate is positioned at the top of the drilling system in this study, peel-up delamination, which typically occurs at the hole entry, was not observed for the CFRP layer. Figure 9 presents the exit zone delamination factor values for each Taguchi trial in detail.

According to the ANOVA results (Table 11), the most influential parameter affecting delamination in the CFRP plate is the feed rate, explaining 46.71% of the total variance (*p* = 0.002). An increase in feed rate causes the tool to remove a larger amount of material per unit time, thereby leading to a rise in thrust force. The increased thrust force leads to stress accumulation at the composite ply–fiber interfaces and results in an increase in push-out delamination. This finding aligns with numerous studies in the literature. For instance, Hocheng and Dhara stated that an increase in feed rate significantly raised the delamination factor [39]. Similarly, Rawat and Attia [40] and Xu et al. [41,42] reported that high feed rates exacerbated ply separation in CFRP layers.

**Figure 10:**
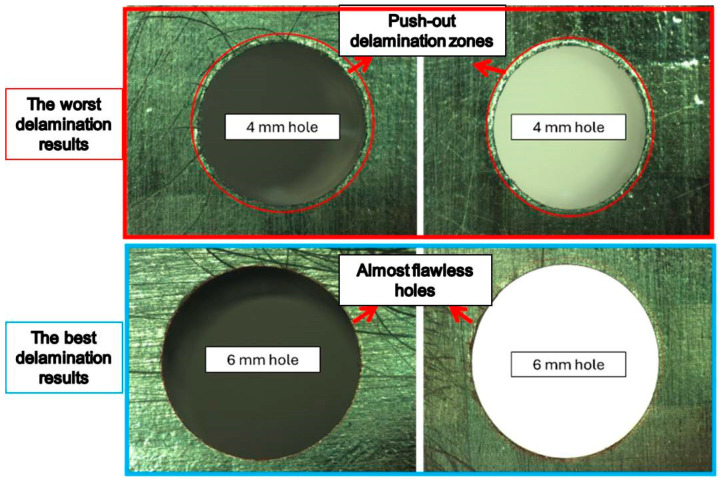
The worst and the best delamination responses on CFRP exit holes.

The second most influential parameter was the drill diameter, with a contribution ratio of 35.00% (*p* = 0.0008). Increasing the diameter enlarges the contact area with the cutting edge, generating higher cutting loads and causing the expansion of the delamination zone, particularly at the exit region. This situation leads to an increase in the delamination factor. The coating condition exhibited a limited effect, with a contribution ratio of 3.93% (*p* = 0.101). The reason for the lack of significance of this parameter is that, although the coating delays tool wear by reducing friction, delamination is predominantly associated with variations in macro-force levels. Additionally, this situation can be attributed to the combined influence of the bending effect at the exit side of the hole and rising frictional forces associated with plastic deformation in the fiber sections. As the same cutting geometry was used in the tools and the point angle was kept constant, cutting mechanics showed a similar trend, and the effect of the coating condition remained relatively low. The contribution ratio of the cutting speed is 6.60% (*p* = 0.205), which was not found to be statistically significant. At high cutting speeds, the temperature rise at the fiber–matrix interface causes resin softening, which complicates the delamination behavior [43]. Overall, it was observed that the most influential parameters on delamination for the CFRP plate were the feed rate and drill diameter. The direct correlation between the delamination mechanism and the thrust force supports the determinant role of these two parameters. In Figure 10, damage images obtained from OM analyses conducted at the hole exit of the CFRP side are presented, specifically for the best- and worst-performing samples. The pronounced effect of the drill diameter on push-out delamination is also evident in these damage analyses.

**Figure 11:**
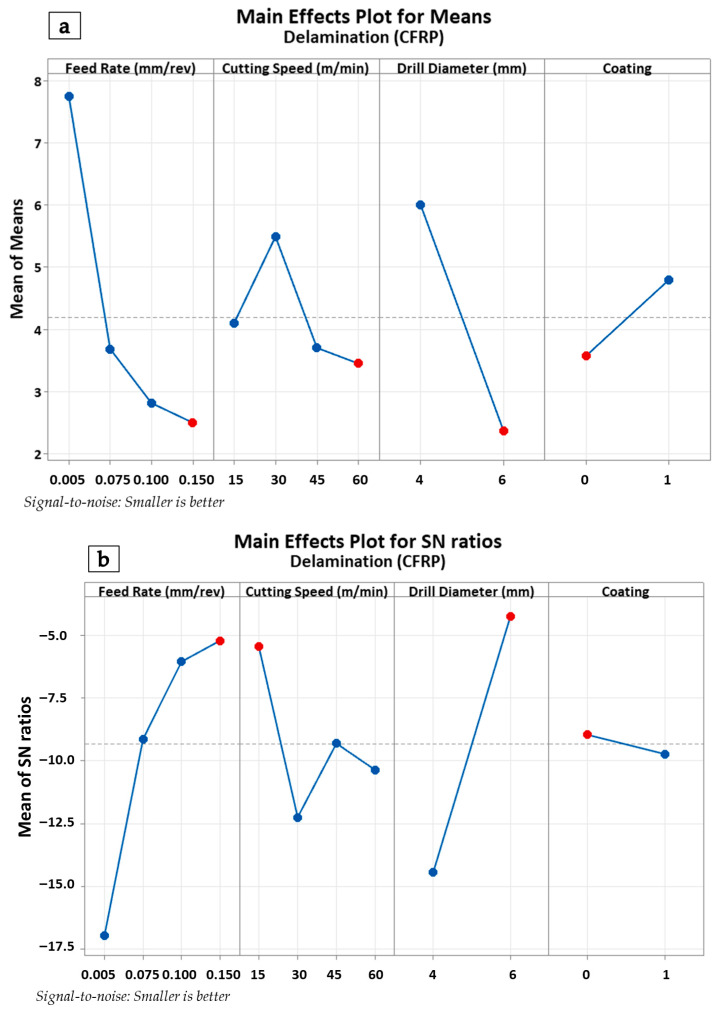
Main effects plot for the CFRP plate (**a**) and S/N ratio plot for the CFRP plate (**b**).

The main effects and S/N plots presented in Figure 11a,b indicate that feed rate and drill diameter have the most pronounced effects on delamination in the CFRP layer (Delta ranking: Feed Rate > Drill Diameter > Cutting Speed > Coating). A distinct reduction in delamination values was observed as the feed rate was increased from 0.050 mm/rev to 0.150 mm/rev. This phenomenon can be explained by the fact that at low feed rates, the tool pulls and breaks the fibers, causing an increase in thrust force and a rise in delamination, whereas, with increased feed rate, the cutting mechanism becomes more stable, resulting in reduced delamination. Upon examining the drill diameter, it was observed that higher delamination values were obtained with the 4 mm tool, while the delamination level dropped significantly with the 6 mm tool. The reason for this is that in small-diameter tools, the short cutting edge triggers fiber–matrix separation, leading to delamination. Cutting speed was observed to have a fluctuating but limited effect on CFRP delamination within the 15–60 m/min range. Regarding the coating condition, the use of coated tools exhibited a slight tendency to increase delamination levels compared to uncoated tools; however, it was determined that the coating did not provide any significant improvement in delamination.

**Figure 12:**
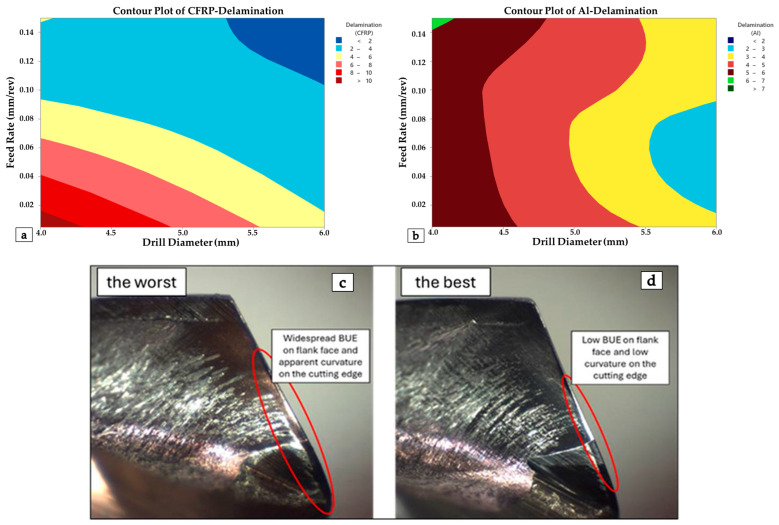
Contour plots for (**a**) delamination in CFRP, (**b**) damage factor in Al plates, and OM views of drill bits utilized for (**c**) the worst and (**d**) the best CFRP delamination outcomes.

The contour plots presented in Figure 12a,b illustrate the variation in damage factor and delamination factor levels for CFRP and Al layers as a function of drill diameter and feed rate. As observed in the plots, a high risk of delamination exists for CFRP laminated structures at small drill diameters and low feed rates. Conversely, the risk of delamination can be minimized with large drill diameters combined with feed rates of 0.1 mm/rev and above. On the other hand, to reduce the hole periphery damage factors in metal plates, low to medium feed levels should be selected and, if possible, combine them with large-diameter tools. OM images of the drills yielding the best and worst delamination results in the CFRP layers are presented in Figure 12c. Herein, significant cutting-edge wear behaviors and a pronounced BUE effect are observed, particularly at low feed rates and small drill diameters. With the increase in drill diameter, less BUE and rounding behavior is detected on the flank face of the tool.

## 4. Conclusions

In this study, the effects of drilling parameters (feed rate, cutting speed, drill diameter, and coating condition) on the drilling force and delamination were systematically investigated. It can be proposed that low feed rates and small drill tools should be chosen for lower thrust forces. The effect of feed rate was more apparent for Al sections with a 71% contribution according to statistical evaluations. Cutting speed and coating condition showed a lower effect on the cutting forces of hybrid stacks. Push-out delamination was observed with decreasing tool diameters, and hole quality diminished due to the BUE effect on the cutting edges of the tools. Feed rate (46.7% contribution) and tool diameter (35% contribution) were also the most determinant factors on the delamination factor levels.

Based on these findings, in aerospace manufacturing, drilling-induced forces and delamination directly affect fatigue life, joint integrity, and assembly quality of critical components such as fuselage panels, wing skins, stringers, ribs, and lap joints. The identification of feed rate and drill diameter as the dominant parameters for both Al and CFRP layers provides a clear guideline for process optimization in industrial drilling operations. For instance, in aircraft fuselage assembly, where thousands of fastener holes are drilled in Al–CFRP stacks, controlling feed rate can significantly reduce thrust force and CFRP delamination, thereby minimizing rework, scrap rates, and the need for secondary repairs. The observed reduction in drilling force and improved process stability with coated drills is particularly relevant for high-volume aerospace production lines, where tool life consistency and dimensional accuracy are critical to meeting strict tolerance requirements. Moreover, the limited influence of cutting speed suggests that aerospace manufacturers can prioritize productivity-oriented cutting speeds without substantially increasing drilling forces, as long as feed rate and tool geometry are carefully optimized. In the context of unmanned aerial vehicles (UAVs) and space structures, where lightweight CFRP components are often joined with aluminum frames, the findings highlight the importance of feed rate control to prevent push-out delamination, which could compromise load transfer efficiency and structural reliability. Additionally, the negligible effect of coating on metallic delamination, but its positive role in force reduction, suggests that tool coatings should be primarily selected for enhancing tool durability and process stability rather than for damage mitigation in aluminum layers. Overall, these results provide practical, data-driven insights for aerospace drilling process design, enabling manufacturers to achieve higher quality joints, extended tool life, and more stable machining conditions in safety-critical applications. From a quality-cost perspective, the reduction in CFRP delamination through feed rate control minimizes costly post-processing steps such as composite patch repairs, local reinforcements, or component rejection, all of which significantly increase manufacturing expenses in aerospace environments.

## Figures and Tables

**Table 1:** Drilling parameters and their levels used in the experiments.

Symbol	Drilling Parameter	Levels			
		1	2	3	4
A	Drill Diameter (mm)	4	6	−	−
B	Feed Rate (mm/rev)	0.05	0.075	0.10	0.15
C	Cutting Speed (m/min)	15	30	45	60
D	Coating Condition (−)	0	1	−	−

**Table 2:** Taguchi Orthogonal Design (L16 (4^2^2^2^)).

Trial No	Feed Rate (mm/rev)	Cutting Speed (m/min)	Drill Diameter (mm)	Coating Condition (0 = Uncoated) (1 = Coated)
1	0.05	15	4	1
2	0.05	30	4	1
3	0.05	45	6	0
4	0.05	60	6	0
5	0.075	15	4	0
6	0.075	30	4	0
7	0.075	45	6	1
8	0.075	60	6	1
9	0.1	15	6	1
10	0.1	30	6	1
11	0.1	45	4	0
12	0.1	60	4	0
13	0.15	15	6	0
14	0.15	30	6	0
15	0.15	45	4	1
16	0.15	60	4	1

**Table 3:** Properties of the resin polymer.

Property	Result
Density	1.28 g/cm^3^
Glass transition temperature	185 °C
Elongation at failure (flexural)	5%
Flexural yield strength	147 MPa
Flexural modulus	3.5 GPa

**Table 4:** Properties of the carbon fibers.

Property	Result
Tensile strength	4646 MPa
Tensile modulus	231 GPa
Elongation at failure	1.8%
Density	1.78 g/cm^3^
Weight/length ratio	0.4 g/m
Filament diameter	6.9 micron
Carbon content	94%

**Table 5:** Some significant features of the utilized drill tools.

Property Group	Parameter	Value/Information
Base tool material	Material type	Sintered carbide
	Composition	90% WC + 10% Co
	Standard type	ISO K20–K40
	Grain size	0.5–0.8 µm
	Hardness	1630 HV30
	Density	14.42 g/cm^3^
Coating material	Coating type	TiAlN
	Coating technique	Physical vapor deposition (PVD)
	Thickness	3 µm
	Hardness	3000 HV
	Max. service temperature	1100 °C
	Coefficient of friction	0.4–0.6 (dry steel reference)

**Table 6:** Model summary for aluminum layers.

S	R-sq	R-sq(adj)	PRESS	R-sq(pred)	AICc	BIC
9.45474	99.40%	98.72%	3269.20	96.87%	168.07	131.79

**Table 7:** ANOVA for aluminum layers.

Source	DF	Seq SS	Contribution	Adj SS	Adj MS	*p*-Value
Feed Rate (mm/rev)	3	74,497	71.22%	74,496.7	24,832.2	0.0000001224
Cutting Speed (m/min)	3	1120	1.07%	1120.4	373.5	0.0544214129
Drill Diameter (mm)	1	27,425	26.22%	27,425.0	27,425.0	0.0000004868
Coating Condition	1	932	0.89%	931.9	931.9	0.0144756415
Error	7	626	0.60%	625.7	89.4	
Total	15	104,600	100.00%			

**Table 8:** Model summary for CFRP layers.

S	R-sq	R-sq(adj)	PRESS	R-sq(pred)	AICc	BIC
4.41199	98.99%	97.83%	711.888	94.71%	143.68	107.40

**Table 9:** ANOVA evaluation for CFRP layers.

Source	DF	Seq SS	Contribution	Adj SS	Adj MS	*p*-Value
Feed Rate mm/rev)	3	5890.2	43.77%	5890.2	1963.41	0.0000040108
Cutting Speed (m/min)	3	167.3	1.24%	167.3	55.77	0.1136164982
Drill Diameter (mm)	1	7075.7	52.58%	7075.7	7075.67	0.0000002718
Coating Condition	1	186.3	1.38%	186.3	186.30	0.0174781772
Error	7	136.3	1.01%	136.3	19.47	
Total	15	13,455.7	100.00%			

**Table 10:** ANOVA for delamination in the Al plate.

Source	DF	Seq SS	Contribution	Adj SS	Adj MS	F-Value
						*p*-Value
Feed Rate (mm/rev)	3	1.2609	3.02%	1.2609	0.4203	0.38
						0.768
Cutting Speed (m/min)	3	3.2216	7.72%	3.2216	1.0739	0.98
						0.455
Drill Diameter (mm)	1	29.5664	70.88%	29.5664	29.5664	27.00
						0.001
Coating Condition	1	0.0004	0.00%	0.0004	0.0004	0.00
						0.985
Error	7	7.6644	18.37%	7.6644	1.0949	
Total	15	41.7137	100.00%			

**Table 11:** Analysis of Variance (ANOVA) for delamination in the CFRP plate.

Source	DF	Seq SS	Contribution	Adj SS	Adj MS	*p*-Value
Feed Rate (mm/rev)	3	70.467	46.71%	70.467	23.489	0.0023935993
Cutting Speed (m/min)	3	9.954	6.60%	9.954	3.318	0.2049778717
Drill Diameter (mm)	1	52.804	35.00%	52.804	52.804	0.0007991185
Coating Condition	1	5.921	3.93%	5.921	5.921	0.1018798264
Error	7	11.703	7.76%	11.703	1.672	
Total	15	150.849	100.00%			

## Data Availability

The data that support the findings of this study are available from the corresponding author upon reasonable request.
